# Author Correction: The oldest known lepidosaur and origins of lepidosaur feeding adaptations

**DOI:** 10.1038/s41586-025-09782-6

**Published:** 2025-11-10

**Authors:** Daniel Marke, David I. Whiteside, Thitiwoot Sethapanichsakul, Robert A. Coram, Vincent Fernandez, Alexander Liptak, Elis Newham, Michael J. Benton

**Affiliations:** 1 School of Earth Sciences, University of Bristol, Bristol, UK; 2 School of Geosciences, Grant Institute, University of Edinburgh, Edinburgh, UK; 3 Fossil Reptiles, Amphibians and Birds Section, The Natural History Museum, London, UK; 4 Department of Earth Sciences, University College London, London, UK; 5 European Synchrotron Radiation Facility, Grenoble, France; 6 Diamond Light Source, Harwell Science and Innovation Campus, Didcot, UK; 7 School of Engineering and Materials Science and Institute of Bioengineering, Queen Mary University of London, London, UK

**Keywords:** Palaeontology, Phylogenetics

Correction to: *Nature* 10.1038/s41586-025-09496-9 Published online 10 September 2025

In the version of this article initially published, the Data availability section was incomplete and did not list further data on phylogenetic data sets and analyses, as well as 3D segmented models from CT scans, available at 10.5061/dryad.cvdncjth4. The changes have been made in the HTML and PDF versions of the article.

