# Supplementary material for: The oldest known lepidosaur and origins of lepidosaur feeding adaptations
Source: Nature. 2025 Sep 10;647(8090):663–72. doi: 10.1038/s41586-025-09496-9 (PMC12629995; doi:10.1038/s41586-025-09496-9)
Supplement: Supplementary file 1 — Additional description of the specimen. [file 41586_2025_9496_MOESM1_ESM.pdf]

nature portfolio

https://doi.org/10.1038/s41586-025-09496-9

Supplementary information

The oldest known lepidosaur and origins of lepidosaur feeding adaptations

In the format provided by the
authors and unedited

S1. Description skull, comparative references Maxillae of *Gephyrosaurus* (see ref. [16], fig. 2). Typical triradiate form of postorbital in lepidosaurs (see ref. [16], fig. 2).
Jugal in the basal rhynchocephalians (e.g. *Planocephalosaurus*, see ref. [20], fig. 1 a, c) and clevosaurs (see ref. [21], fig. 4D; [22], fig. 12A), and the pan lepidosaurs *Marmoretta* and *Paliguana* (see ref. [11], fig. 4).
Ectopterygoid as seen in *Clevosaurus hudsoni* and *C. cambrica* see ref. [21], fig. 8D).
The parasbasisphenoid strongly resembles that in *Sphenodon* (see ref. [27], fig. 3.1).
The coronoid process is not as high or pointed as found in the Late Triassic *Clevosaurus hudsoni*, *C. cambrica* (see ref.[21], fig. 10) or *Clevosaurus brasiliensis*, more resembling *Microsphenodon* (see ref.[29], fig. 8) and *Sphenodon* (see ref.[6], fig. 1.21 D, E).
This suite of dental features can be observed in clevosaurids like *C. hadroprodon* (see ref.[30], fig 2).

In the **Discussion of phylogenetic characters**, the olfactory processes are similar to those of *Clevosaurus* (see ref. [42], fig. 5f). Ford et al.[11] consider *Sophineta*, which has a conch, as a lepidosaur whereas we find it outside the clade.

The teeth in *Agriodontosaurus* differ from those of *Diphydontosaurus* where the anterior teeth are positioned medially of the crista dorsalis and the posterior teeth are positioned apically; the opposite is observed in *Agriodontosaurus*.

Description of BRSUG 29950-14 postcranial elements.

Vertebrae. A sequence of eight cervical vertebrae is preserved in articulation (Figs. 2A and Extended Data Fig. 1) posterior to the skull but the atlas or axis cannot be identified in the scan. The dorsal elements such as neural arches and zygapophyses are absent, either having been lost to abrasion or too small to discern. Detail here is poor, preventing a good reconstruction, but it is possible to make out in cross-section that the cervical vertebrae are amphicoelous and notochordal (Extended Data Fig. 4). This is the condition in closely related taxa such as *Diphydontosaurus*, *Gephyrosaurus*, stem lepidosauromorphs (=pan-lepidosaurs[17]) and basal crown squamates like gekkonomorphans. There are two posterior presacral vertebrae, one of

which shows the dorsal processes including much of the neural arch, zygapophyses and both transverse processes (Extended Data Fig. 5). The pleurocentrum exhibits a pronounced midventral crest and lacks condyles, as is often the case with amphicoelous vertebrae (Extended Data Fig. 5F).

Pectoral Girdle and Forelimbs. The entire pectoral girdle (Extended Data Fig. 6) is preserved, with scapula, coracoids, clavicles and interclavicle in near articulation, with the left-hand elements most complete and the right coracoid and scapula fragmented. The interclavicle is rodlike posteriorly and positioned medially to each coracoid. Although missing the left lateral process, the anterior portion is T-shaped, as observed in pan-lepidosaurs like *Marmoretta* and *Fraxinisaura*, but absent or reduced in derived rhynchocephalians[33]. These lateral processes contact the posterior faces of the clavicles. The posterior process of the interclavicle expands into an unusual bulbous termination not seen in other early lepidosaurs (Extended Data Fig. 6). The *clavicles* (Extended Data Fig. 6) extend laterally from the contact with the ventral face of the interclavicle, following the anterior extent of the coracoids before projecting dorsolaterally along the anterior margins of the scapulae. The clavicles are unornamented by processes, flanges or fenestrations, and they articulate with the dorsal process of the scapula with a flat facet dorsally.

The *coracoids* (Extended Data Fig. 6) are broad and flat, forming most of the ventral region of the pectoral girdle, and they lack a supracoracoid foramen and fenestra; one hole on the right coracoid is considered as damage rather than a fenestra. At their medial contact with the interclavicle, they are relatively thin and thicken toward the lateral margins and the facet for articulation with the scapulae to form a distinct lateral crest when observed ventrally. The contact of the scapula and coracoid forms the glenoid (Extended Data Fig. 6B), and the posterior part of the facet may be seen in a subtle projection from the lateral crest of the coracoid.

The *scapula* (Extended Data Fig. 6) is rectangular, projecting dorsolaterally from the facet of the coracoid as well as slightly anteriorly. The anterior margin of the scapula is thin and bears a distinct emargination towards its dorsal end, as observed in iguanians and rhynchocephalians. It also lacks a scapular ray. There is no evidence of a supraglenoid foramen, and the thickened lateral margin of the coracoid continues up the posterior margin of the scapula to form the supraglenoid buttress that can be seen in dorsal view (Extended Data Fig. 6A).

Both *humeri* (Extended Data Fig. 7) are preserved, but the right humerus is incomplete, whereas the left is more complete and closely associated with the pectoral girdle. The proximal end is well preserved, the deltopectoral crest is visible and connected to the rest of the humeral head. The proximal head is broad in lateral view (Extended Data Fig. 7B, D) and makes approximately a third of the total length of the humerus, which then tapers to a straight shaft and expands at the distal end, which is oriented perpendicular to the proximal end. The distal end of the left humerus is damaged but many of the important features are preserved. In lateral view, there is a distinct ectepicondylar foramen (Extended Data Fig. 7A, B) like those seen in derived squamates and rhynchocephalians[5,56]. The ectepicondyles are expanded and separated from the entepicondyles by a groove-like supinator process (Extended Data Fig. 7A). The entepicondyles lack a distinct foramen and are much less expanded than their lateral counterparts, not exceeding the medial extent of the humeral shaft. The distalmost parts of the condyles for articulation with the ulna and radius are not preserved. Several fragments may represent the *radius* and *ulna* (Fig. 2), but these are uncertain.

Pelvic Girdle and Hindlimbs. All elements of the pelvic girdle, except the right pubis, are preserved but largely crushed and disarticulated. The left *ilium* (Extended Data Fig. 8A–D) is complete and displays all processes for articulation with the other pelvic elements. The distinct, rod-like preacetabular process contributes to a distinct supra-acetabular buttress (Extended Data Fig. 8A) on the lateral surface of the ilium, forming the dorsal margin of a closed acetabulum. The iliac blade is broad and curves slightly laterally, narrowing posterodorsally and has a notch on the anterior iliac crest (Extended Data Fig. 8A, C). The posterior contact of the iliac blade with the main body of the ilium shows a distinct notch posterior to the margin of the acetabulum. Notably, the preacetabular process lacks the anterior pubic process seen in most squamates and early reptiles[5,18]. In fact, the ilium resembles the basal rhynchocephalians *Gephyrosaurus bridensis* (see ref. [77], fig. 21) and *Planocephalosaurus robinsonae* (see ref. [78], fig.17). The right *pubis* (Extended Data Fig. 8E, F) is preserved and is not fused with the ischium, a common feature among reptiles, except captorhinids[5]. The pubis is largely rectangular, preserving the ventral margin of the acetabulum in the form of a dorsal concavity. There is no distinct obturator foramen, which is surprising as it is common in limbed reptiles (including rhynchocephalians),

therefore it is likely that it was small and not visible at this resolution. The facets for articulation of the ilium and ischium are preserved. The ventral end of the pubis lacks a pubic tubercle but bears an anterior facet that may be for articulation of an epipubis or represents the pubic symphysis (Extended Data Fig. 8E).

Both *ischia* are complete (Extended Data Fig. 8G), though they have been crushed. Though disarticulated from the other elements, we may infer from other reptiles that the ischia were not fused to the pubes. Further, the anterior margin of the ischium possesses a distinct concavity forming the thyroid fenestra as seen in all but the most basal diapsids. At the midline of the anterior margin, there is a small concavity likely to receive the proischiadic cartilage. The posterior margin shows two protrusions that could represent the hypoischium. There is also a distinct ischiadic tuberosity on the left ischium. Each exhibits a thickening of the lateral margins for articulation with the corresponding ilia, and the left lateral margin also preserves the ischial contribution to the acetabulum in the form of a subtle facet.

Each *femur* (Extended Data Fig. 9) is preserved, but only the left is complete, with the right missing both proximal and distal ends. The internal trochanter of the left femur is preserved alongside a distinct posterior flange on the proximal head and, as with all lepidosaurs, the femur lacks a fourth trochanter (Extended Data Fig. 9D). The medial face of the proximal femur also preserves a subtle intertrochanteric fossa (Extended Data Fig. 9D) that is only absent in a few archosauromorphs like *Euparkeria* and *Tanystropheus* as well as crown squamates with reduced limbs[5,31,33]. The distal region of the femur preserves both anterior and posterior condyles for articulation with the distal limb bones, divided by a distinct popliteal fossa (Extended Data Fig. 9B).

Of the distal hindlimb elements, only a *tibia* and *fibula* are preserved, the former lacking both its proximal and distal ends but retaining what appears to be a facet to receive the proximal end of the *fibula*. The incomplete nature of these elements makes them largely uninformative (Fig.
2).

77. Evans, S. E. The postcranial skeleton of the Lower Jurassic eosuchian *Gephyrosaurus bridensis*. *Zool. J. Linn. Soc.* **73**, 81–116 (1981).
78. Fraser, N. C. & Walkden, G. M. The postcranial skeleton of the Upper Triassic sphenodontid *Planocephalosaurus robinsonae*. *Palaeontology* **27**, 575–595 (1984).
