# Supplementary material for: The oldest known lepidosaur and origins of lepidosaur feeding adaptations
Source: Nature. 2025 Sep 10;647(8090):663–72. doi: 10.1038/s41586-025-09496-9 (PMC12629995; doi:10.1038/s41586-025-09496-9)
Supplement: Supplementary file 2 — Reporting Summary [file 41586_2025_9496_MOESM2_ESM.pdf]

nature portfolio

Corresponding author(s): Michael J. Benton

Last updated by author(s): Jun 16, 2025

# Reporting Summary

Nature Portfolio wishes to improve the reproducibility of the work that we publish. This form provides structure for consistency and transparency in reporting. For further information on Nature Portfolio policies, see our Editorial Policies and the Editorial Policy Checklist.

## Statistics

For all statistical analyses, confirm that the following items are present in the figure legend, table legend, main text, or Methods section.

| n/a | Confirmed | |
|---|---|---|
| ☐ | ☒ | The exact sample size (*n*) for each experimental group/condition, given as a discrete number and unit of measurement |
| ☒ | ☐ | A statement on whether measurements were taken from distinct samples or whether the same sample was measured repeatedly |
| ☒ | ☐ | The statistical test(s) used AND whether they are one- or two-sided<br>*Only common tests should be described solely by name; describe more complex techniques in the Methods section.* |
| ☒ | ☐ | A description of all covariates tested |
| ☒ | ☐ | A description of any assumptions or corrections, such as tests of normality and adjustment for multiple comparisons |
| ☒ | ☐ | A full description of the statistical parameters including central tendency (e.g. means) or other basic estimates (e.g. regression coefficient) AND variation (e.g. standard deviation) or associated estimates of uncertainty (e.g. confidence intervals) |
| ☒ | ☐ | For null hypothesis testing, the test statistic (e.g. *F*, *t*, *r*) with confidence intervals, effect sizes, degrees of freedom and *P* value noted<br>*Give P values as exact values whenever suitable.* |
| ☐ | ☒ | For Bayesian analysis, information on the choice of priors and Markov chain Monte Carlo settings |
| ☒ | ☐ | For hierarchical and complex designs, identification of the appropriate level for tests and full reporting of outcomes |
| ☒ | ☐ | Estimates of effect sizes (e.g. Cohen's *d*, Pearson's *r*), indicating how they were calculated |

*Our web collection on statistics for biologists contains articles on many of the points above.*

## Software and code

Policy information about availability of computer code

| | |
|---|---|
| Data collection | Dragonfly v. 2022.2; Avizo 2020.2 |
| Data analysis | Standard phylogenetic analysis tools (PAUP, tnt v.1.6, MrBayes v.3.2.7) and programs in R (ape, paleotree). |

For manuscripts utilizing custom algorithms or software that are central to the research but not yet described in published literature, software must be made available to editors and reviewers. We strongly encourage code deposition in a community repository (e.g. GitHub). See the Nature Portfolio guidelines for submitting code & software for further information.

## Data

Policy information about availability of data

All manuscripts must include a data availability statement. This statement should provide the following information, where applicable:

- Accession codes, unique identifiers, or web links for publicly available datasets
- A description of any restrictions on data availability
- For clinical datasets or third party data, please ensure that the statement adheres to our policy

All data on specimen description and phylogenetic analysis are in the Extended data and SI. In addition, synchrotron X-ray CT data for the characterisation of the skull of Agriodontosaurus helsbypetrae BRSUG 29950-14 are available as raw acquisition data (doi.org/10.15151/esrf-dc-2158672188) and as processed data (doi.esrf.fr/10.15151/ESRF-DC-2160804068). Please cite these as:
Benton, M., & Fernandez, V. (2025). Synchrotron X-ray CT raw data for the characterization of the skull of Agriodontosaurus helsbypetrae BRSUG 29950-14. (Version

1) [Dataset]. European Synchrotron Radiation Facility. doi.org/10.15151/ESRF-DC-2158672188
Benton, M. J., & Fernandez, V. (2025). Synchrotron X-ray CT processed data of the skull of Agriodontosaurus helsbypetrae BRSUG 29950-14. (Version 1) [Dataset]. European Synchrotron Radiation Facility. doi.org/10.15151/ESRF-DC-2160804068
All data on specimen description and phylogenetic analysis are in the Extended data and SI. In addition, synchrotron X-ray CT data for the characterisation of the skull of Agriodontosaurus helsbypetrae BRSUG 29950-14 are available as raw acquisition data (doi.org/10.15151/esrf-dc-2158672188) and as processed data (doi.esrf.fr/10.15151/ESRF-DC-2160804068). Please cite these as:
Benton, M., & Fernandez, V. (2025). Synchrotron X-ray CT raw data for the characterization of the skull of Agriodontosaurus helsbypetrae BRSUG 29950-14. (Version 1) [Dataset]. European Synchrotron Radiation Facility. doi.org/10.15151/ESRF-DC-2158672188
Benton, M. J., & Fernandez, V. (2025). Synchrotron X-ray CT processed data of the skull of Agriodontosaurus helsbypetrae BRSUG 29950-14. (Version 1) [Dataset]. European Synchrotron Radiation Facility. doi.org/10.15151/ESRF-DC-2160804068

## Research involving human participants, their data, or biological material

Policy information about studies with human participants or human data. See also policy information about sex, gender (identity/presentation), and sexual orientation and race, ethnicity and racism.

| | |
|---|---|
| Reporting on sex and gender | N/a |
| Reporting on race, ethnicity, or other socially relevant groupings | N/a |
| Population characteristics | N/a |
| Recruitment | N/a |
| Ethics oversight | N/a |

Note that full information on the approval of the study protocol must also be provided in the manuscript.

# Field-specific reporting

Please select the one below that is the best fit for your research. If you are not sure, read the appropriate sections before making your selection.

☐ Life sciences ☐ Behavioural & social sciences ☒ Ecological, evolutionary & environmental sciences

For a reference copy of the document with all sections, see nature.com/documents/nr-reporting-summary-flat.pdf

# Ecological, evolutionary & environmental sciences study design

All studies must disclose on these points even when the disclosure is negative.

| | |
|---|---|
| Study description | Description of fossil reptile; phylogenetic analysis of morphological traits |
| Research sample | One specimen |
| Sampling strategy | n/a |
| Data collection | Cladistic data matrix taken from Talanda et al. (2022) and modified as described in the Methods. |
| Timing and spatial scale | Phylogenetic data spans from Carboniferous to Recent, and extends spatially worldwide |
| Data exclusions | None |
| Reproducibility | We provide the data matrix and nexus codes for analysis |
| Randomization | n/a |
| Blinding | n/a |

Did the study involve field work? ☐ Yes ☒ No

# Reporting for specific materials, systems and methods

We require information from authors about some types of materials, experimental systems and methods used in many studies. Here, indicate whether each material, system or method listed is relevant to your study. If you are not sure if a list item applies to your research, read the appropriate section before selecting a response.

## Materials & experimental systems

| n/a | Involved in the study |
|---|---|
| ☒ | ☐ Antibodies |
| ☒ | ☐ Eukaryotic cell lines |
| ☐ | ☒ Palaeontology and archaeology |
| ☒ | ☐ Animals and other organisms |
| ☒ | ☐ Clinical data |
| ☒ | ☐ Dual use research of concern |
| ☒ | ☐ Plants |

## Methods

| n/a | Involved in the study |
|---|---|
| ☒ | ☐ ChIP-seq |
| ☒ | ☐ Flow cytometry |
| ☒ | ☐ MRI-based neuroimaging |

# Palaeontology and Archaeology

Specimen provenance: Specimen comes from the Triassic on the Devon coast, on public land

Specimen deposition: Specimen is lodged in University of Bristol geological collections, repository code BRSUG

Dating methods: Published biostratigraphy and magnetostratigraphy data; we did no dating

☐ Tick this box to confirm that the raw and calibrated dates are available in the paper or in Supplementary Information.

Ethics oversight: University of Bristol

Note that full information on the approval of the study protocol must also be provided in the manuscript.

# Plants

Seed stocks: n/a

Novel plant genotypes: n/a

Authentication: n/a
