# Supplementary material for: The oldest known lepidosaur and origins of lepidosaur feeding adaptations
Source: Nature. 2025 Sep 10;647(8090):663–72. doi: 10.1038/s41586-025-09496-9 (PMC12629995; doi:10.1038/s41586-025-09496-9)
Supplement: Supplementary file 3 — Series of vertical slices through the jaws and palate (labelled) from ESRF synchrotron scan data. [file 41586_2025_9496_MOESM3_ESM.pdf]

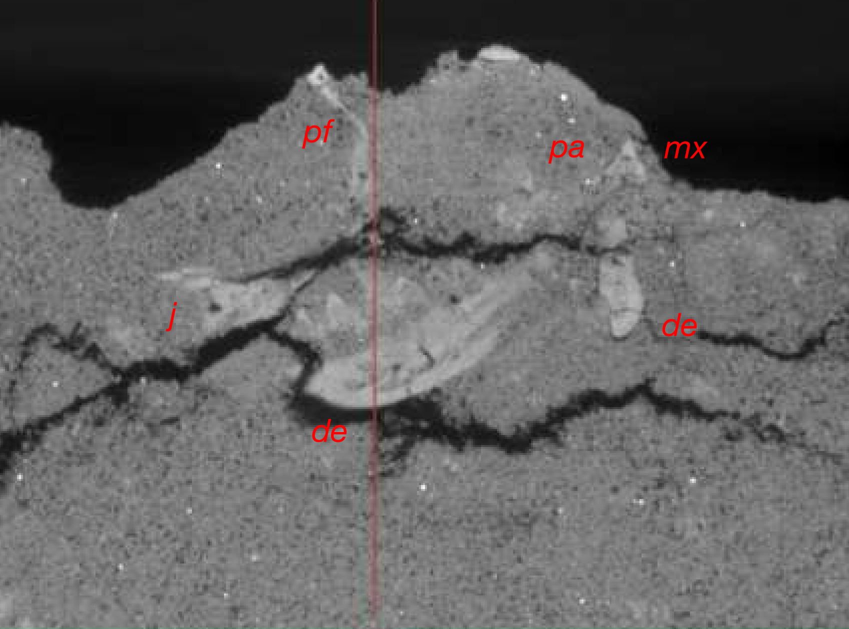
pf
pa
mx
j
de
de

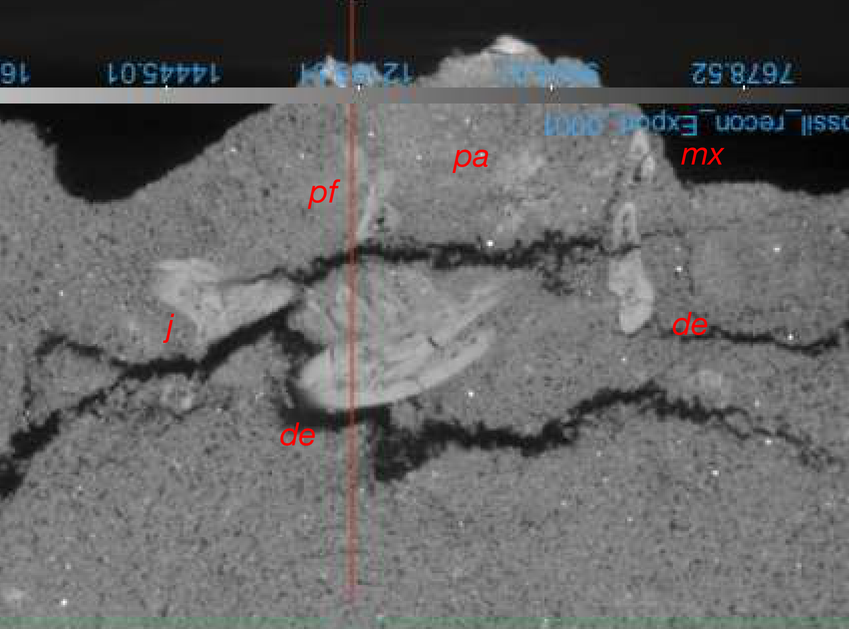
pf
pa
mx
j
de
de

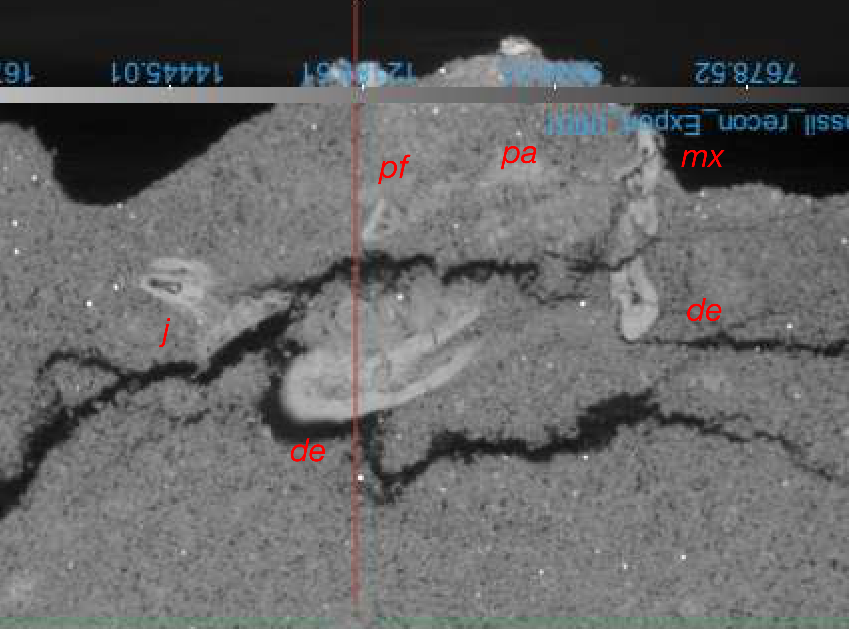
pf
pa
mx
j
de
de
de

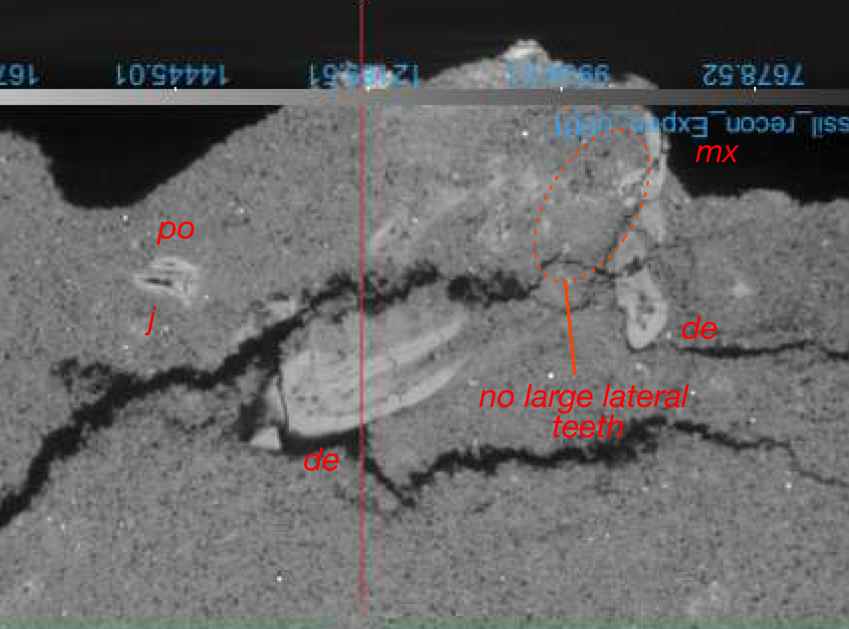
mx
po
j
de
no large lateral teeth
de

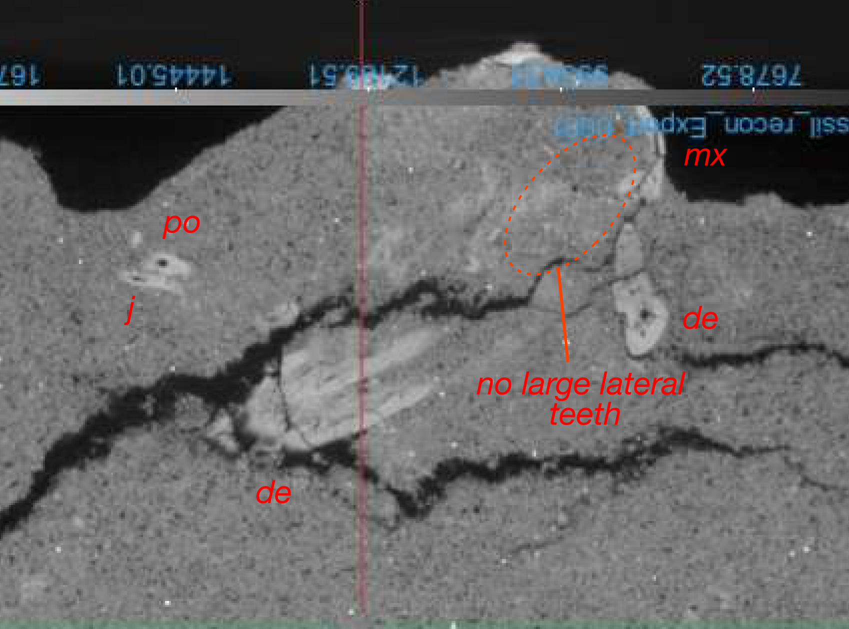

po
j
mx
de
no large lateral teeth
de

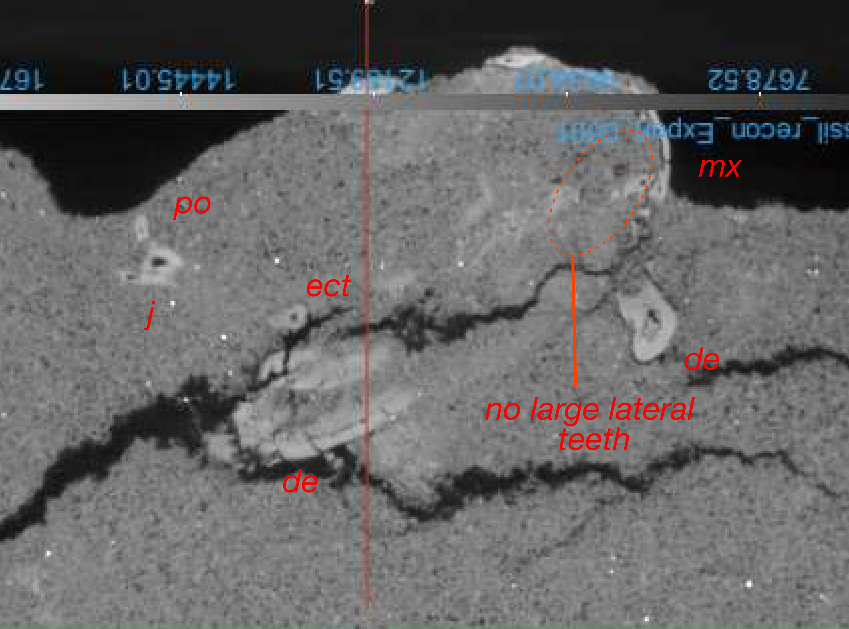
po
j
ect
mx
de
de
no large lateral teeth

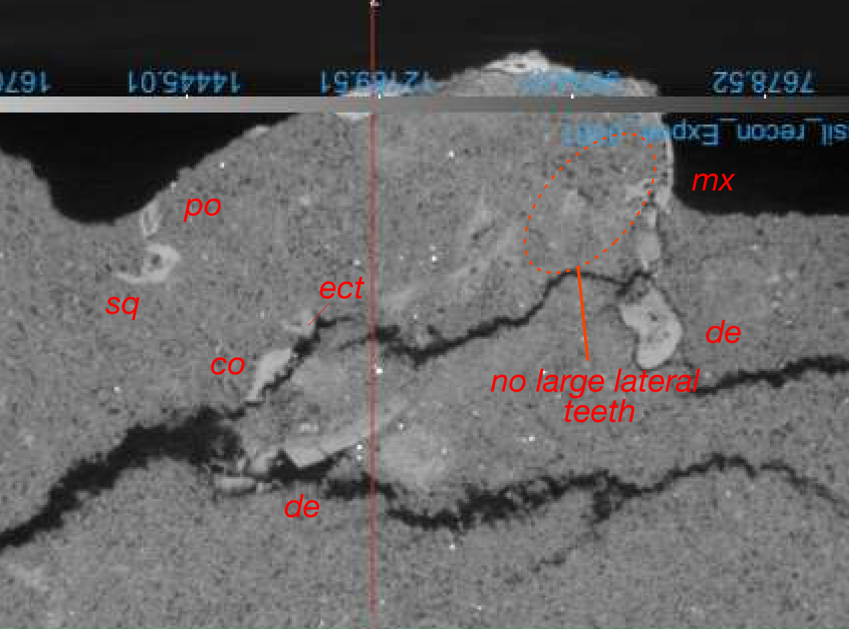
po
mx
sq
ect
co
de
no large lateral
teeth
de

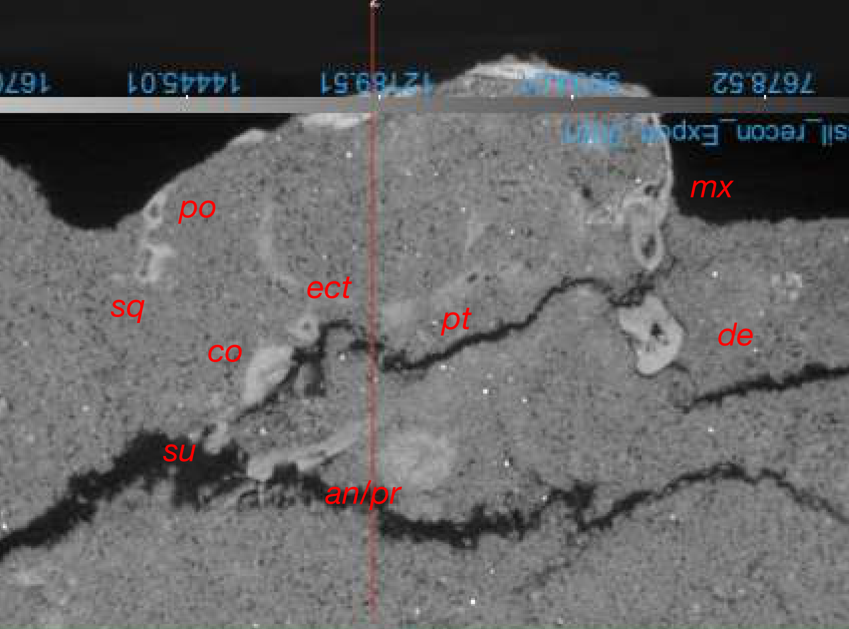
po
mx
ect
sq
pt
co
de
su
an/pr

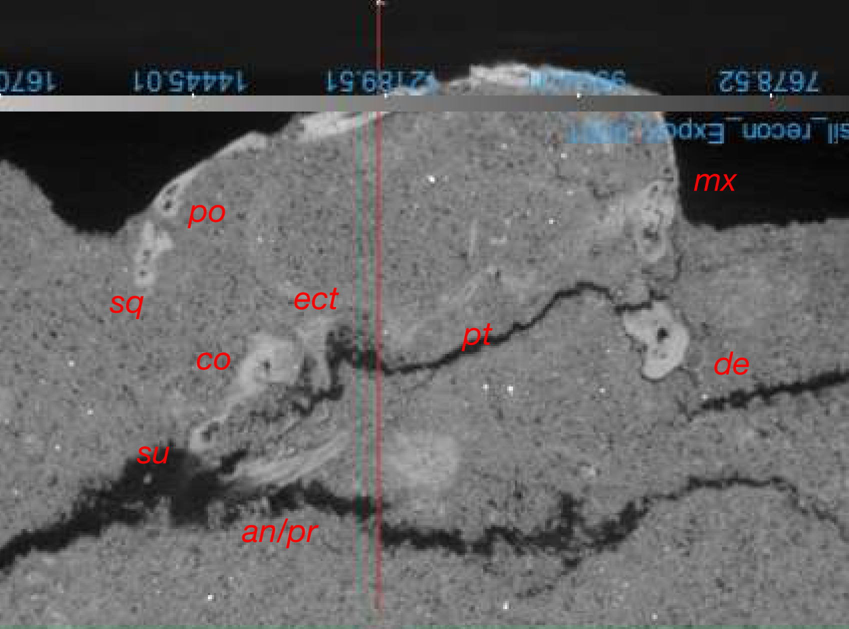
mx
po
sq
ect
pt
co
de
su
an/pr

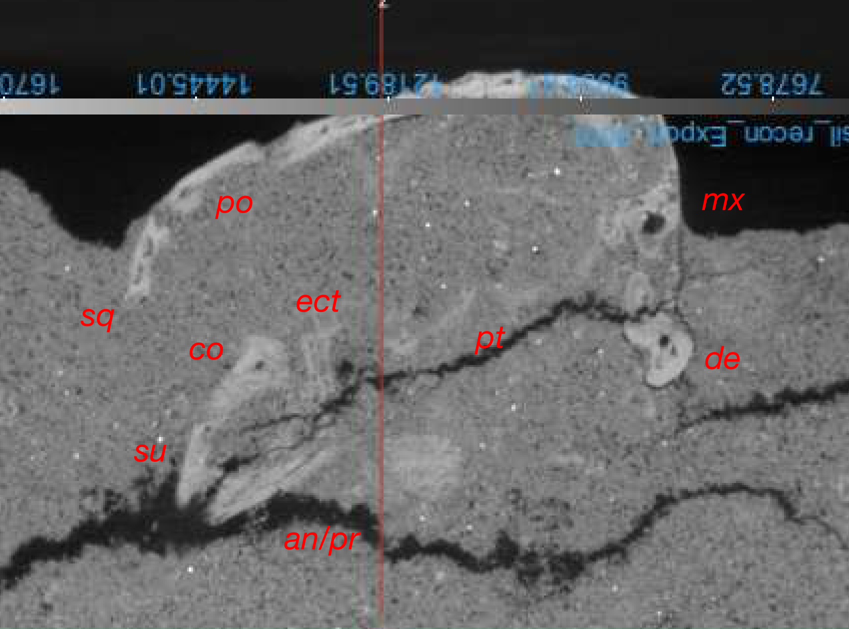
po
mx
sq
ect
co
pt
de
su
an/pr

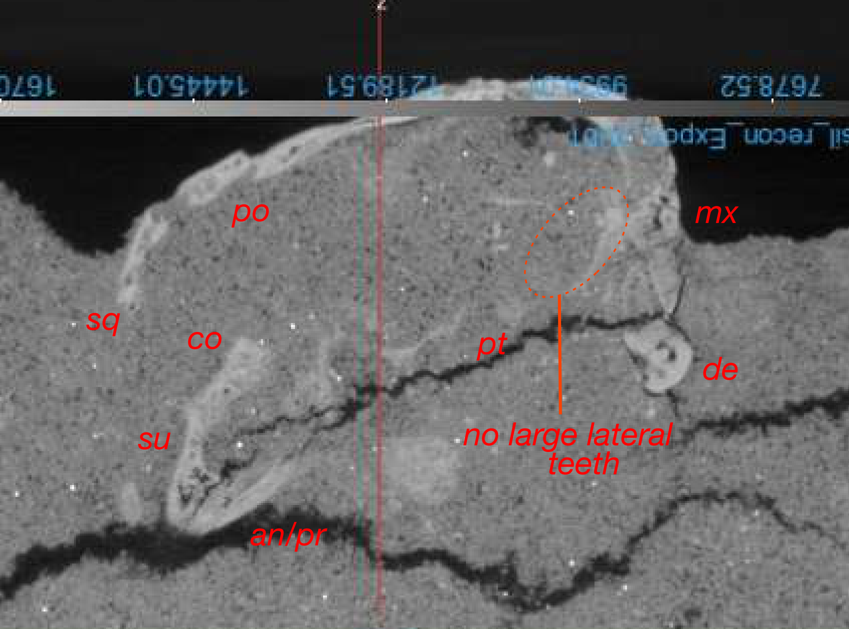
po
mx
sq
co
pt
de
su
no large lateral teeth
an/pr

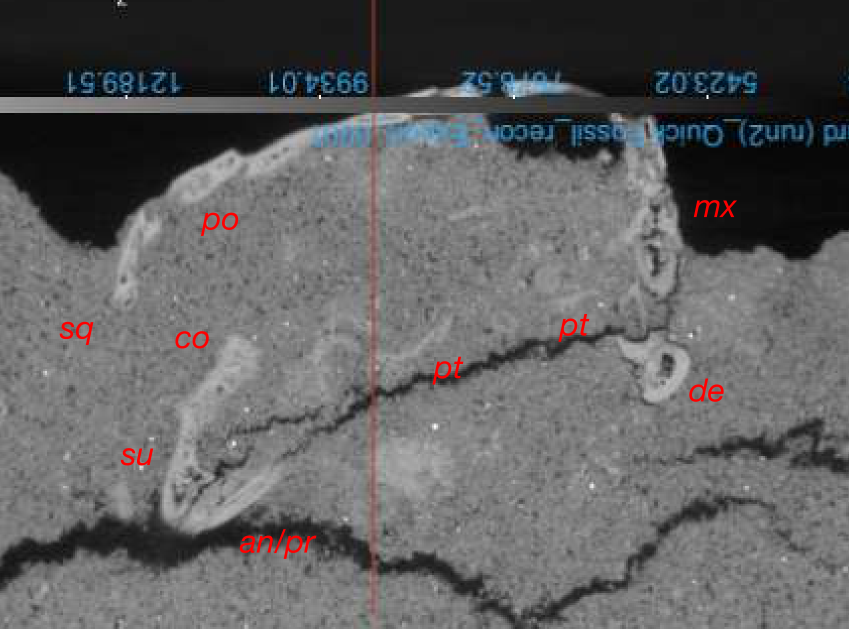
po
mx
sq
co
pt
pt
de
su
an/pr

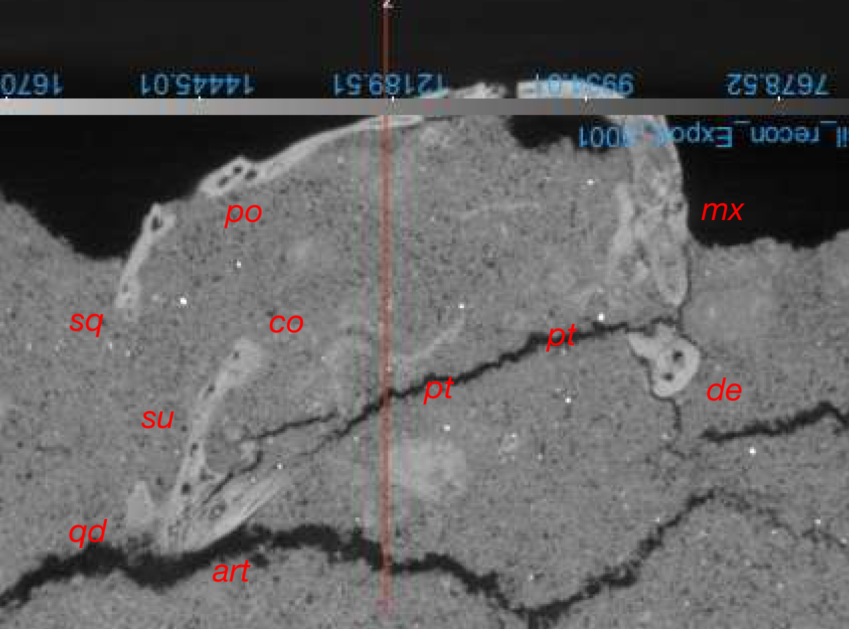
po
mx
sq
co
pt
pt
de
su
qd
art

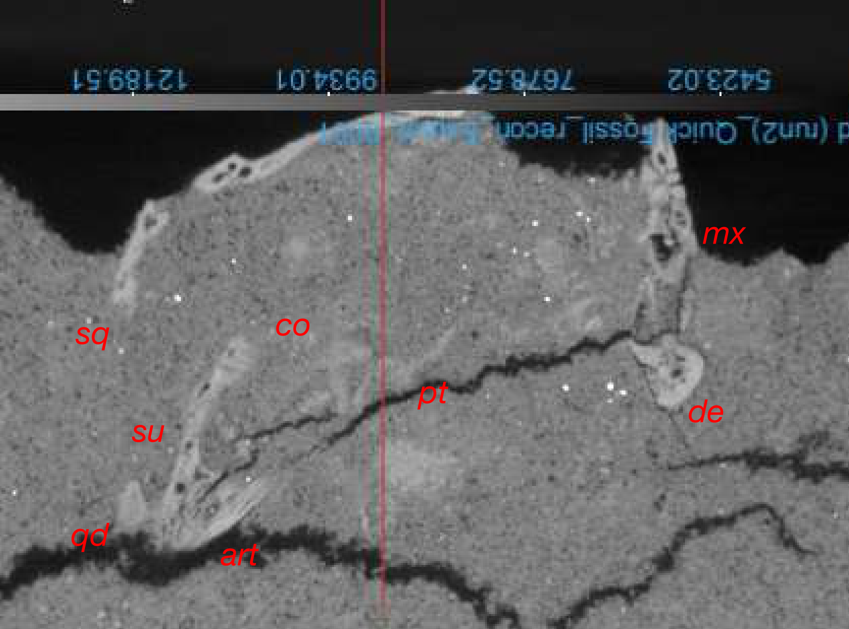
mx
sq
co
pt
de
su
qd
art

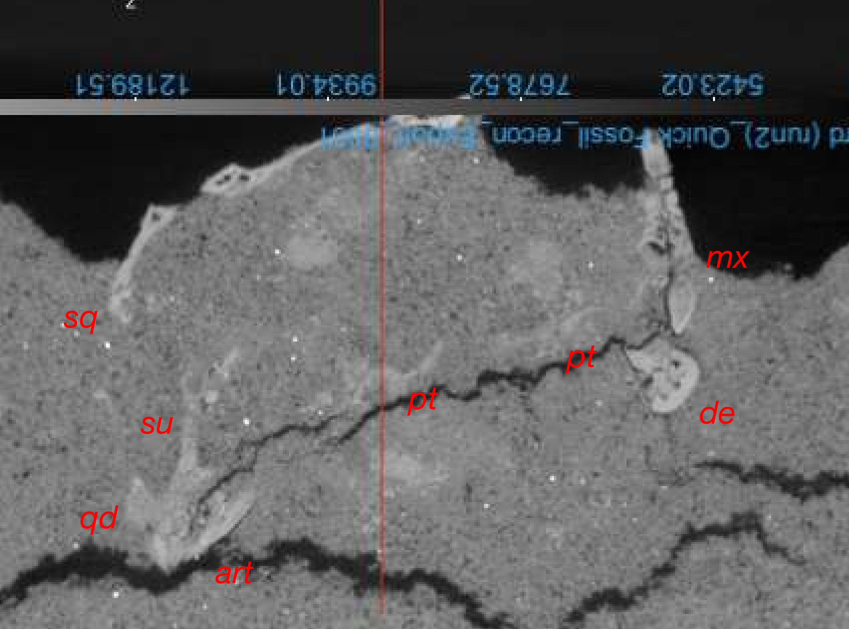
mx
sq
pt
pt
su
de
qd
art

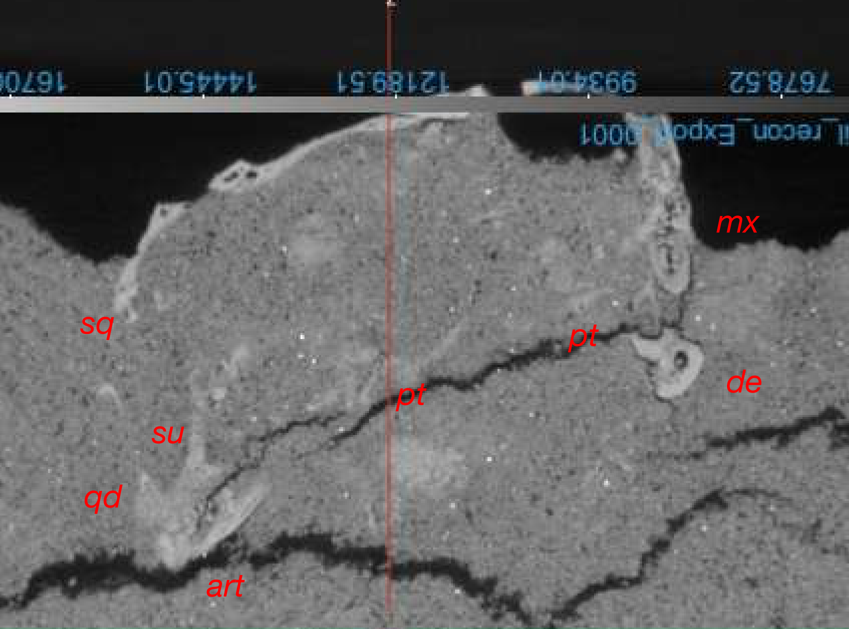
mx
sq
pt
pt
de
su
qd
art

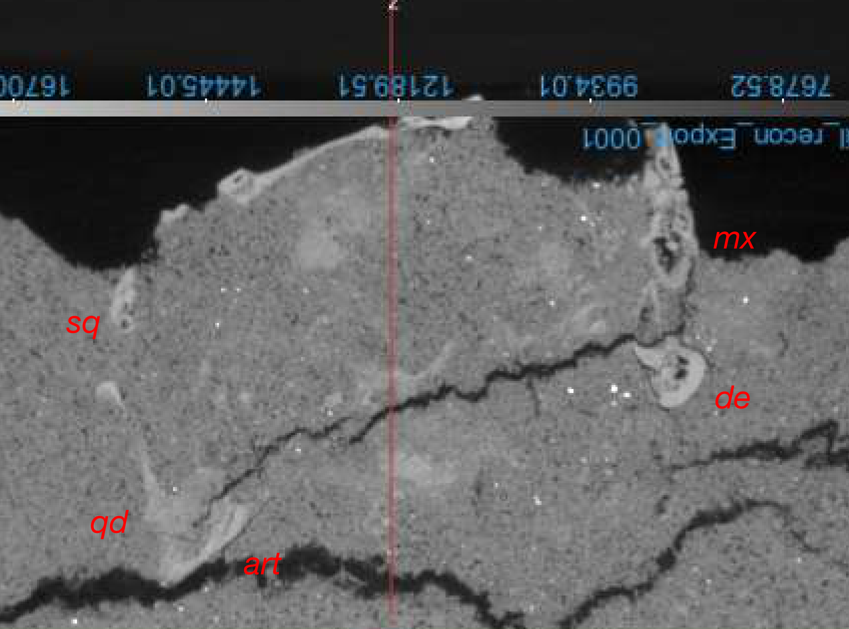
mx
sq
de
qd
art

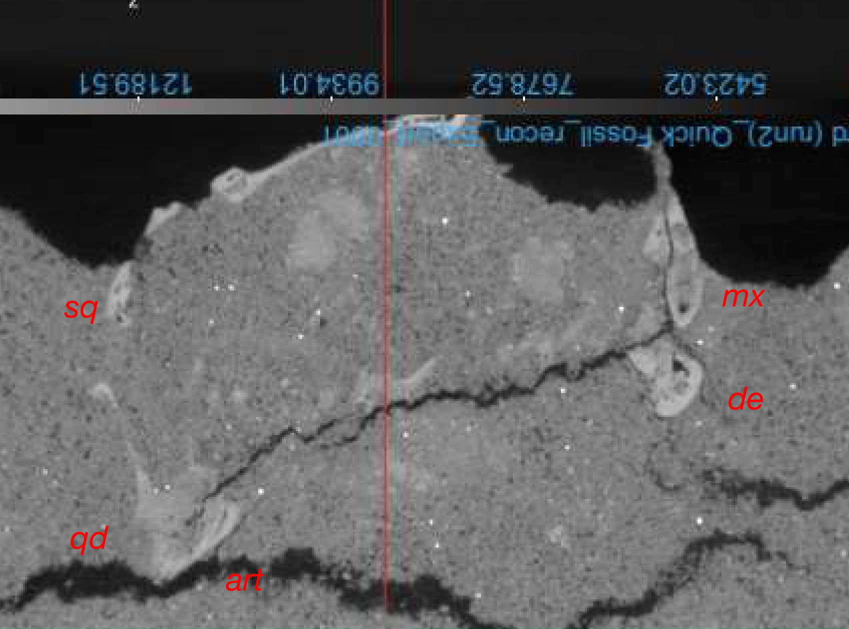
sq
mx
de
qd
art

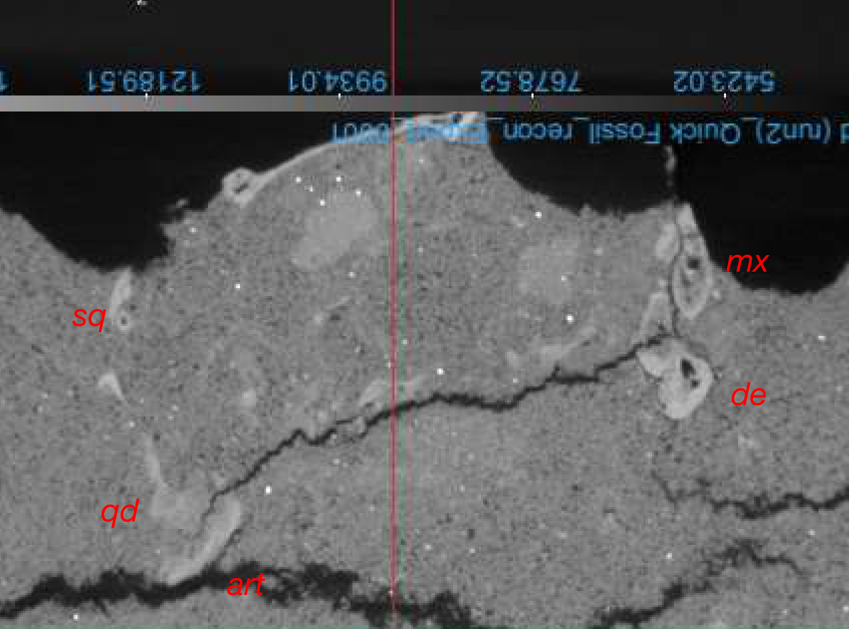
mx
sq
de
qd
art

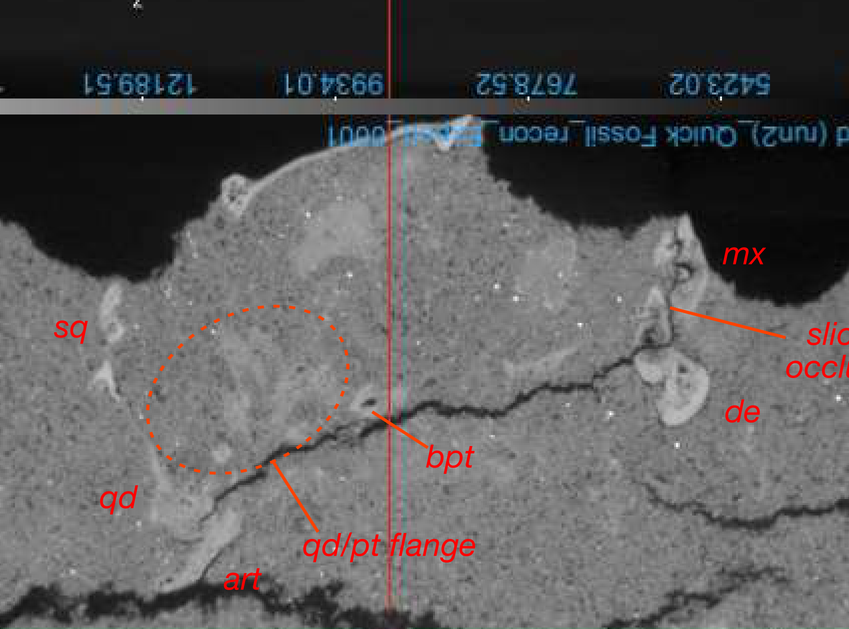
mx
sq
slic
occl
de
bpt
qd
qd/pt flange
art

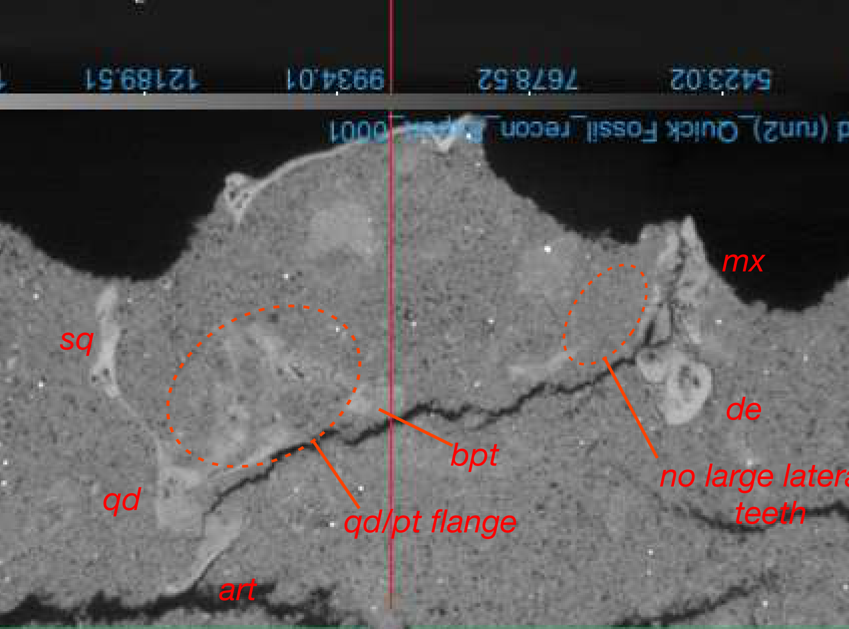
mx
sq
de
bpt
no large latera
teeth
qd
qd/pt flange
art

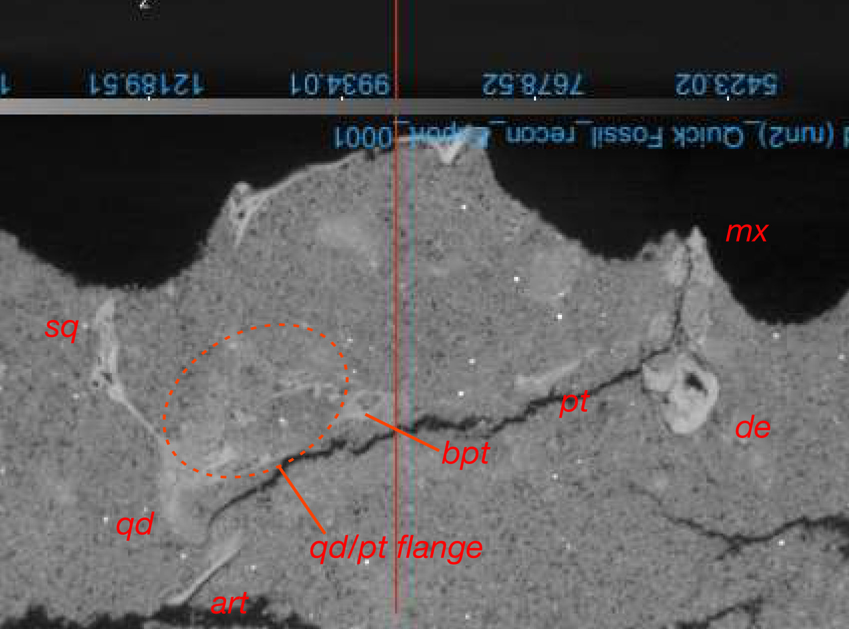

mx
sq
pt
de
bpt
qd
qd/pt flange
art
