# Supplementary material for: The oldest known lepidosaur and origins of lepidosaur feeding adaptations
Source: Nature. 2025 Sep 10;647(8090):663–72. doi: 10.1038/s41586-025-09496-9 (PMC12629995; doi:10.1038/s41586-025-09496-9)
Supplement: Supplementary file 4 — Series of horizontal slices through the jaws and palate (labelled) from Diamond synchrotron scan data. [file 41586_2025_9496_MOESM4_ESM.pdf]

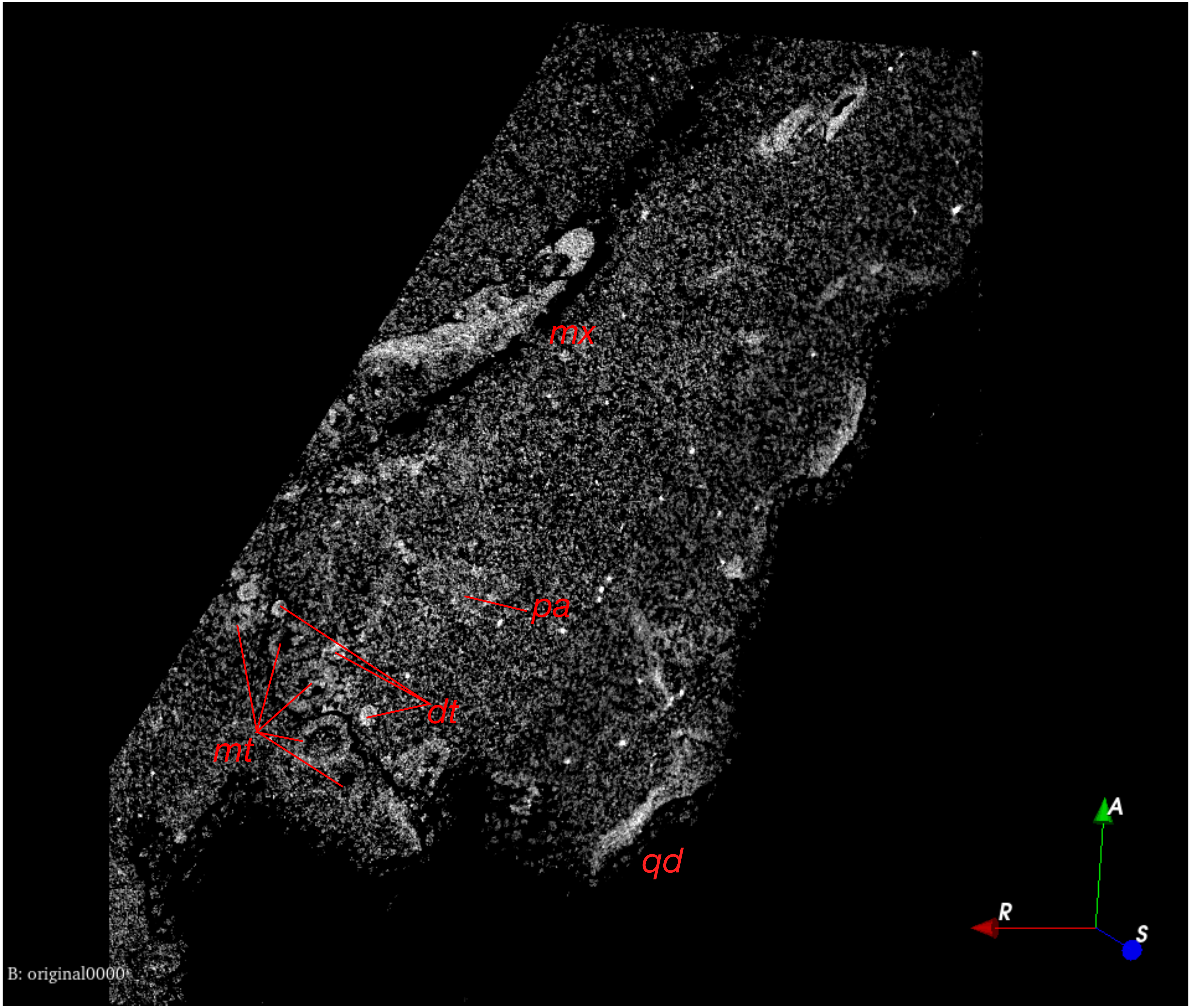
mx
pa
dt
mt
qd
A
R
S
B: original0000

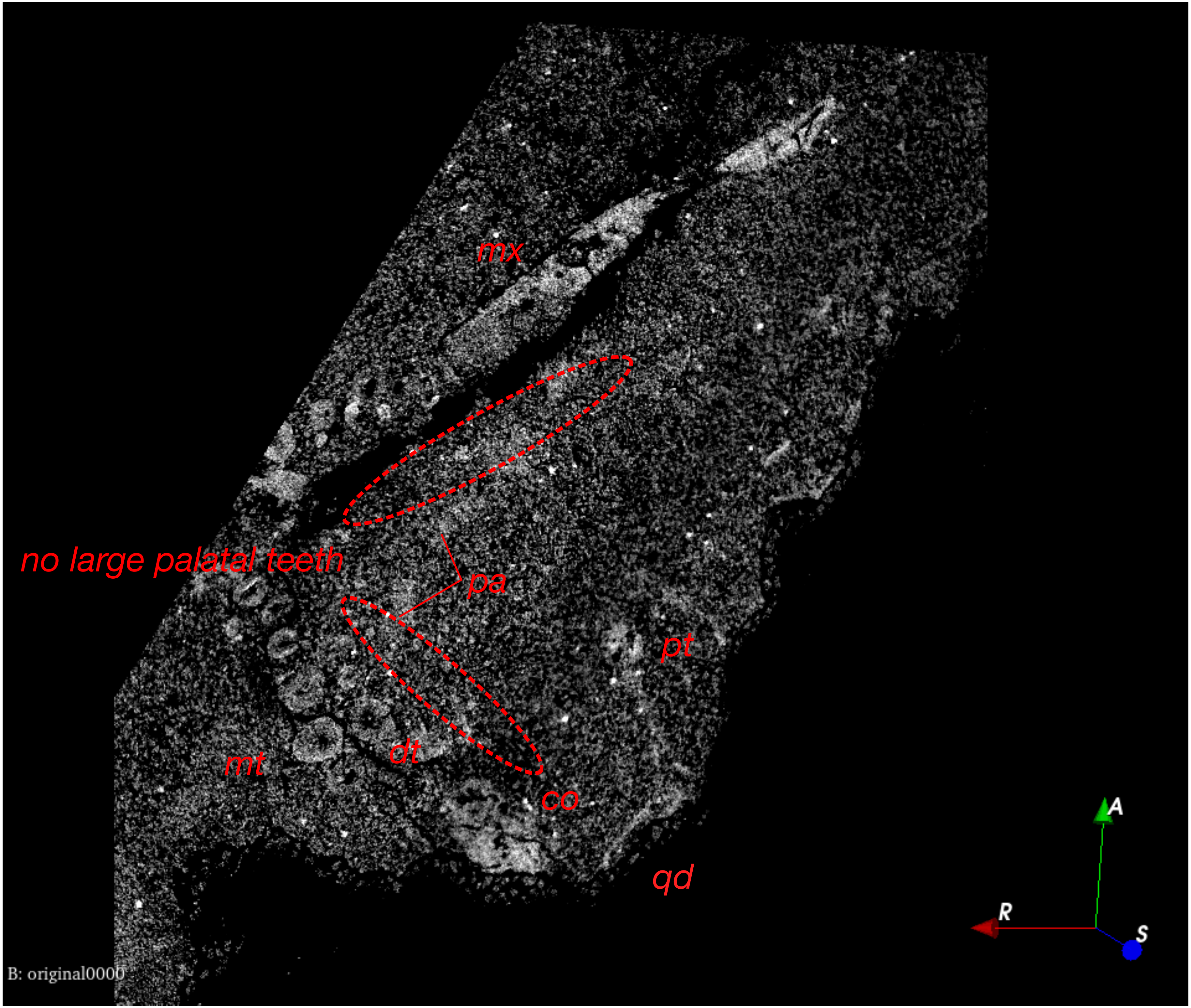

mx
no large palatal teeth
pa
pt
mt
dt
co
qd
A
R
S
B: original0000

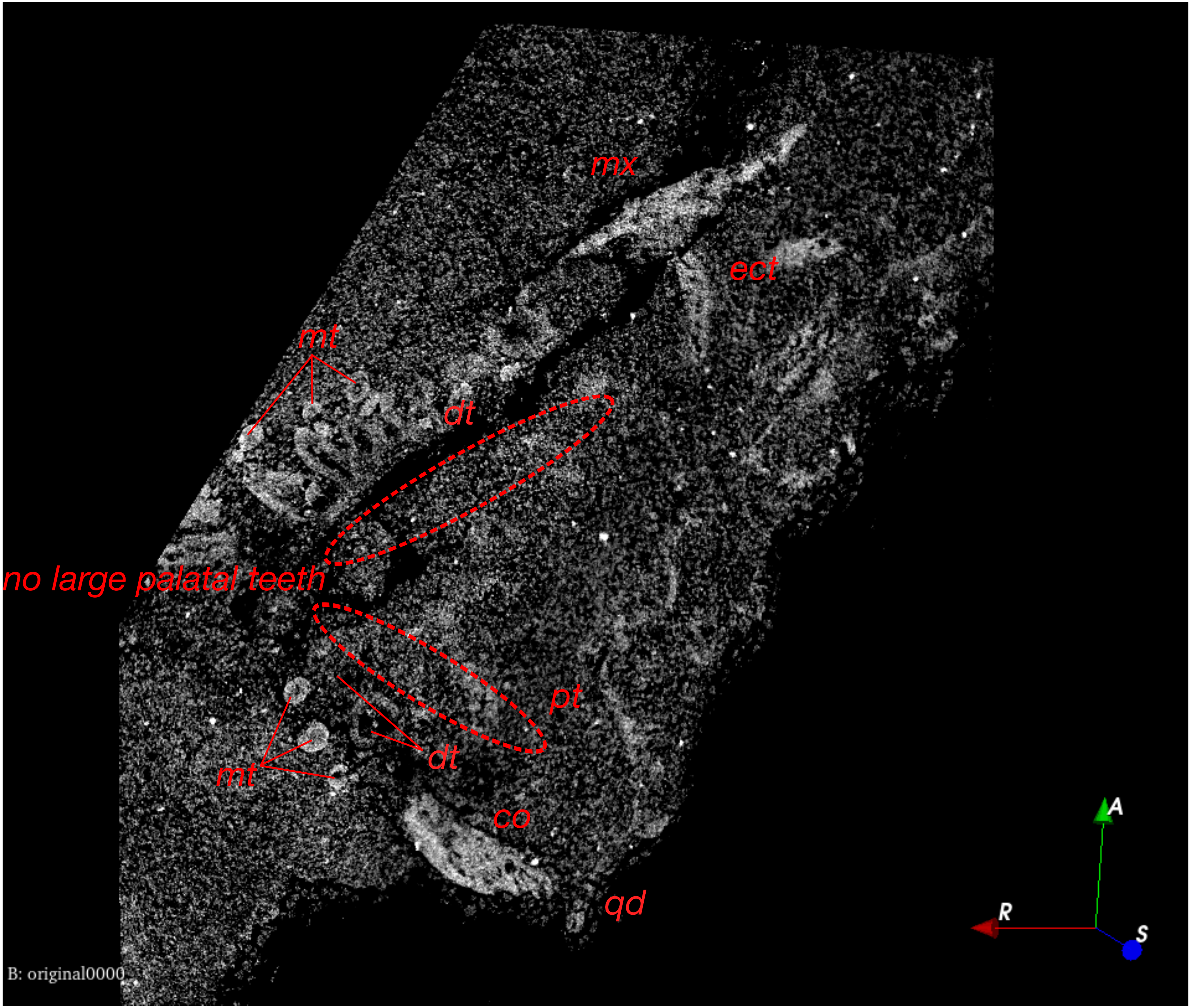
mx
ect
mt
dt
no large palatal teeth
pt
mt
dt
co
qd
A
R
S
B: original0000

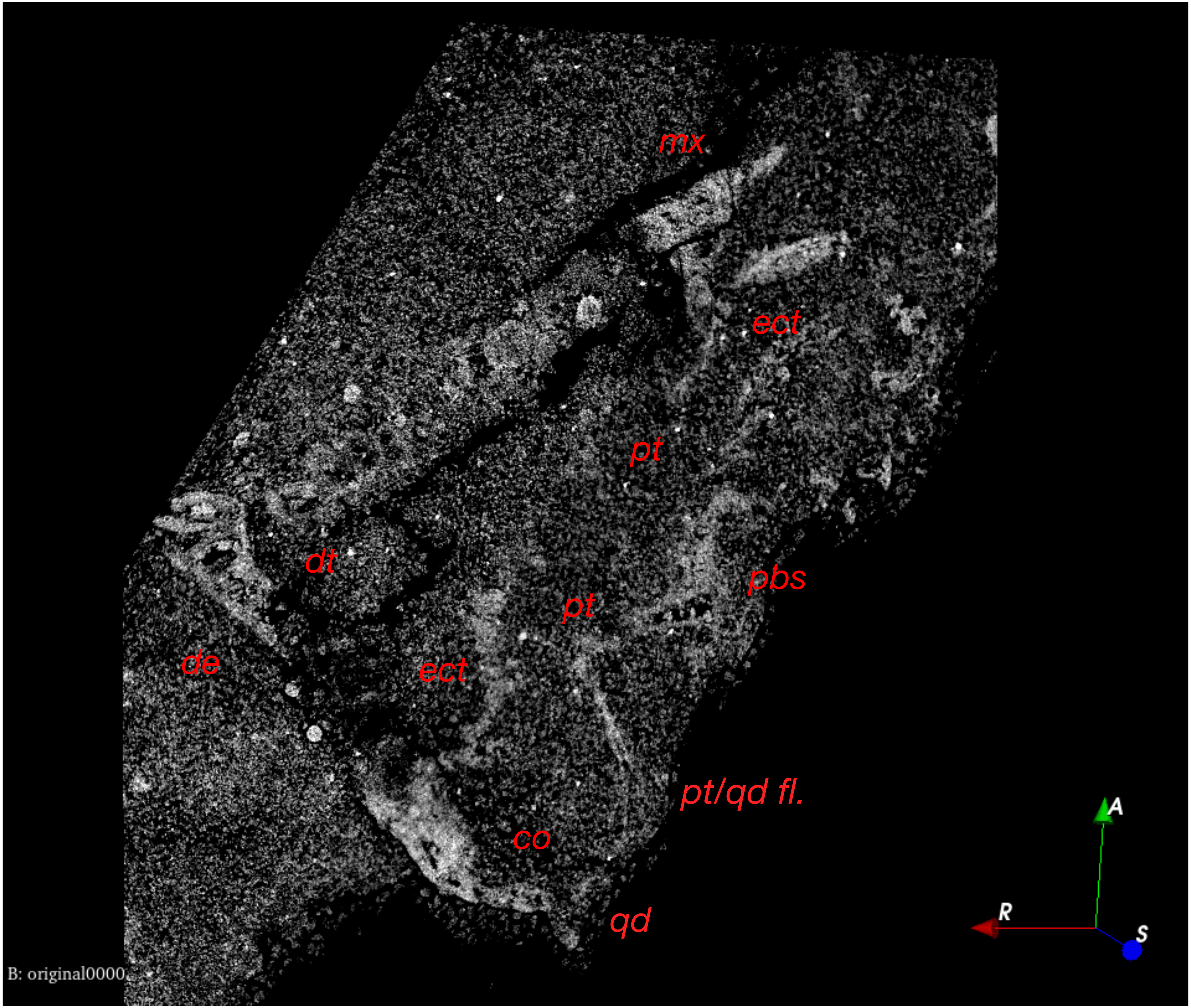
mx
ect
pt
dt
pbs
pt
de
ect
pt/qd fl.
co
qd
A
R
S
B: original0000

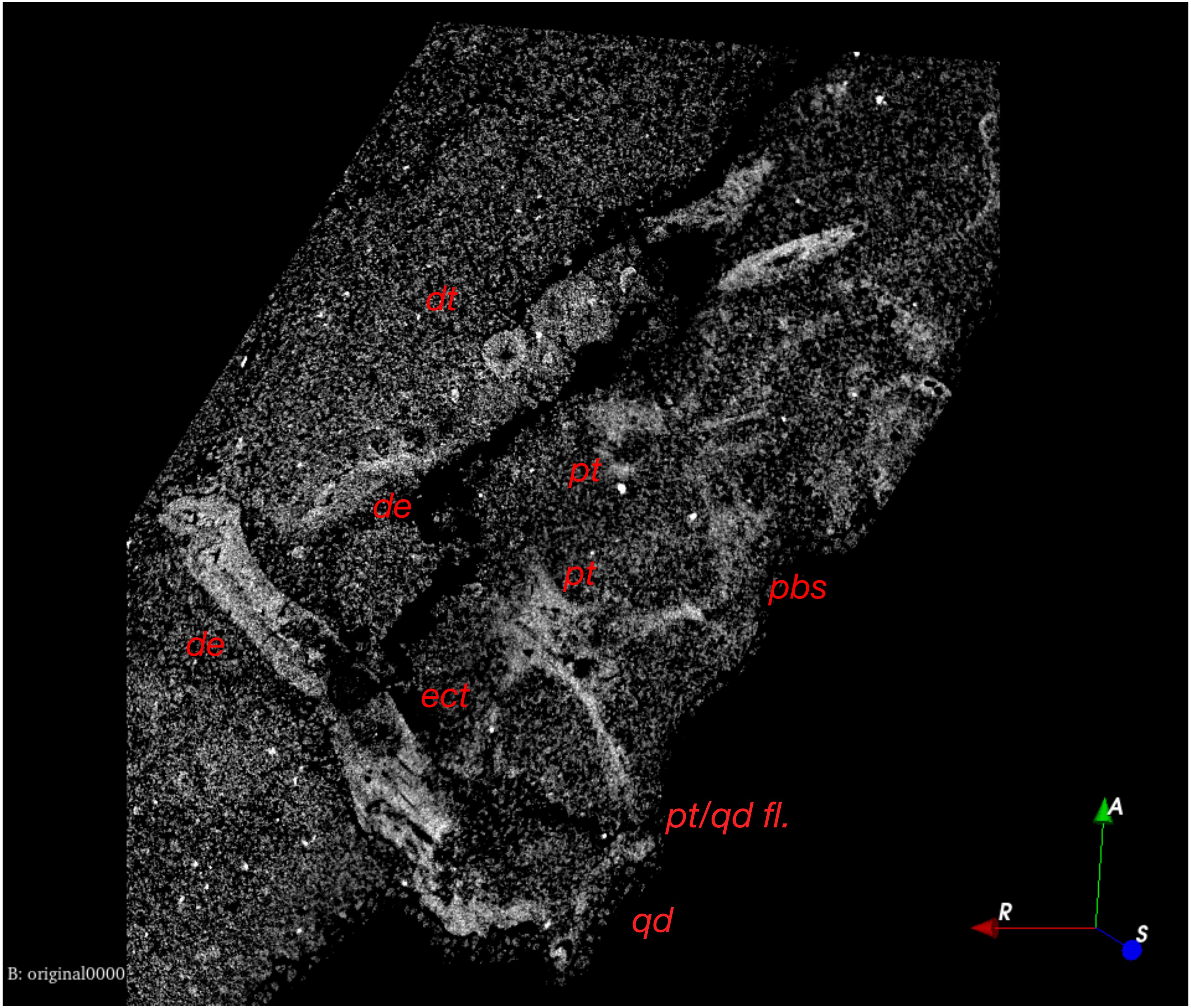
dt
de
pt
pt
pbs
de
ect
pt/qd fl.
qd
A
R
S
B: original0000

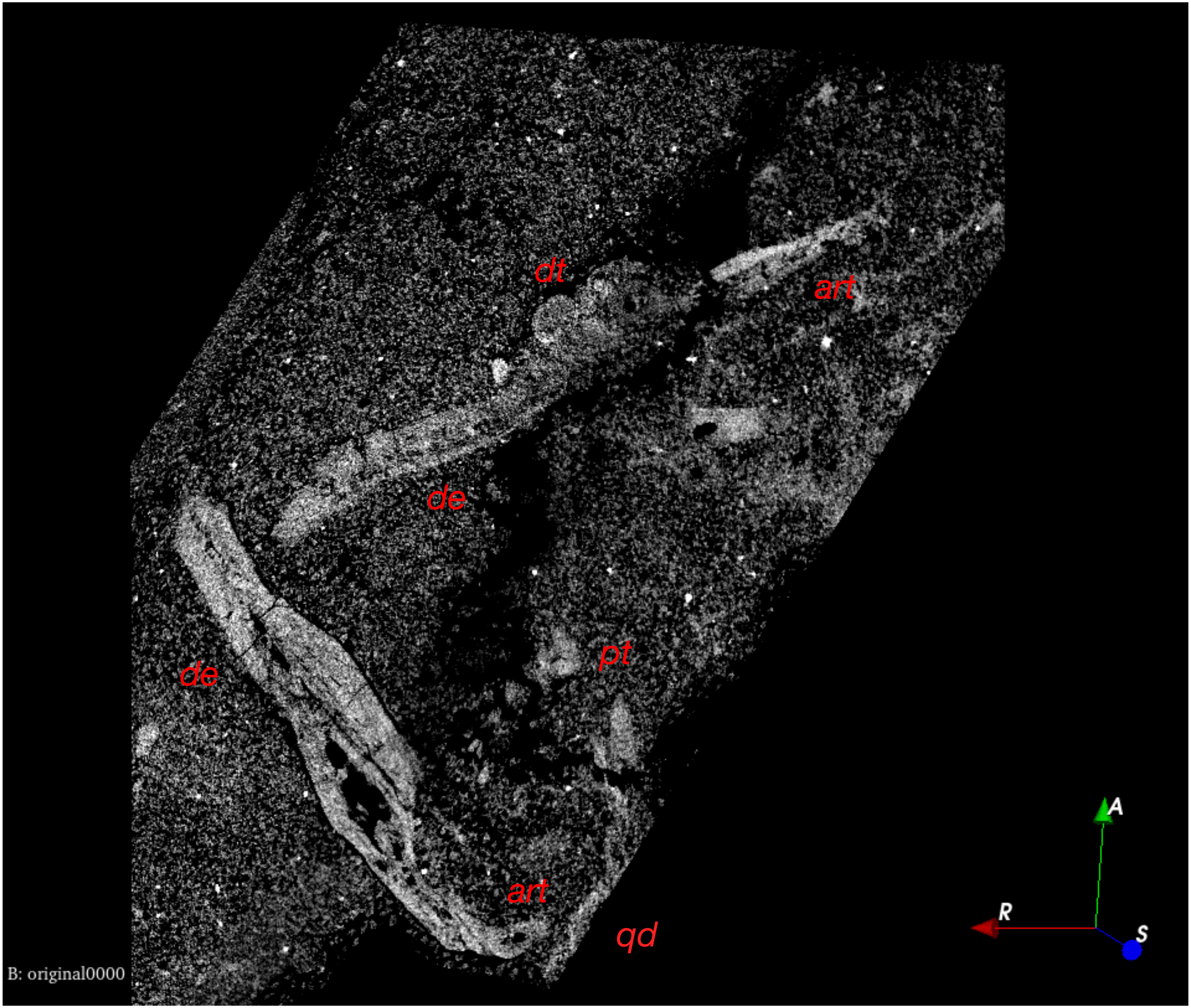
dt
art
de
de
pt
art
qd
A
R
S
B: original0000

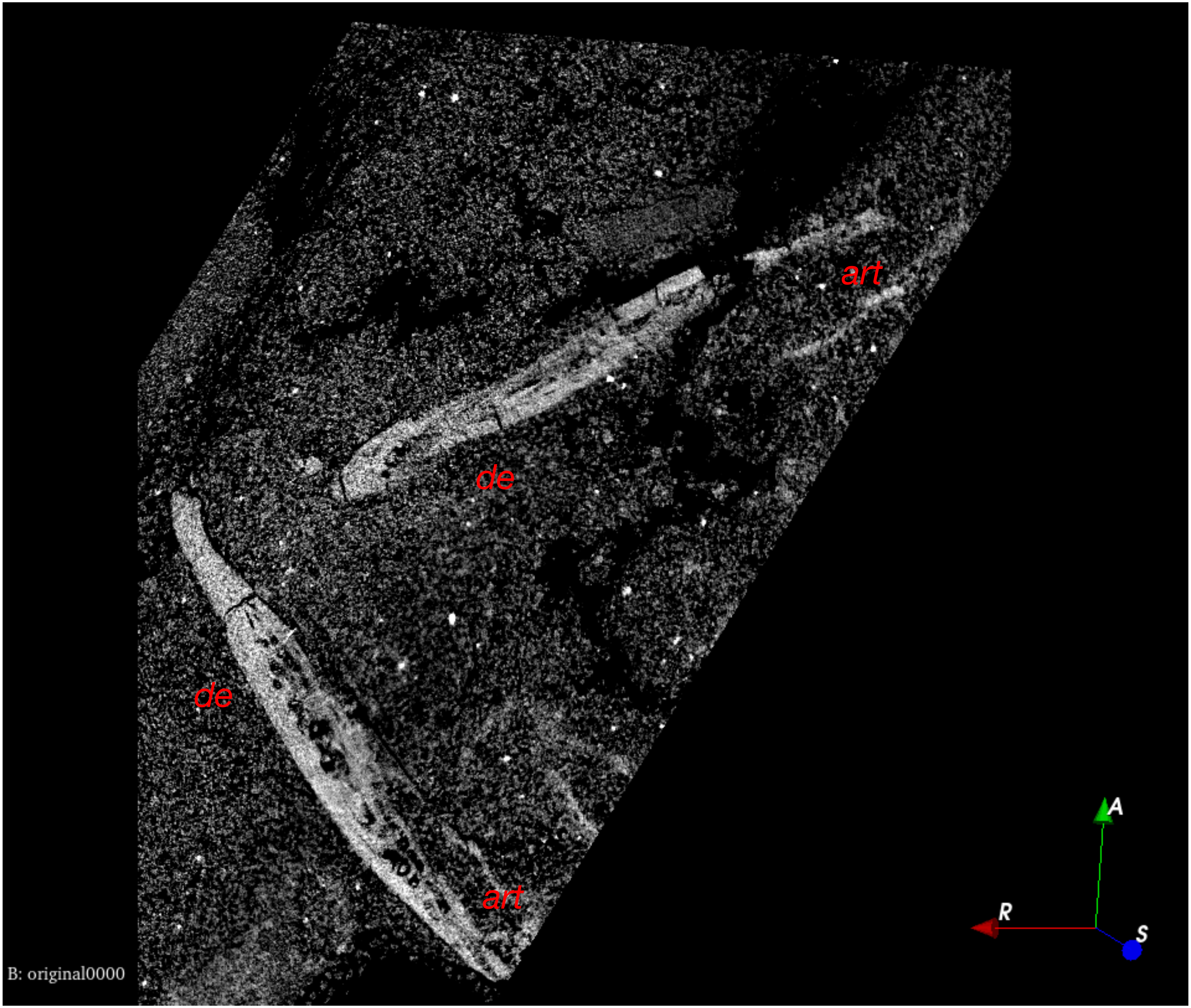
art
de
de
art
A
R
S
B: original0000

Abbreviations:

art, articulars; bpt; co, coronoid; de, dentary; dt, dentary teeth; ect, ectopterygoid; mx, maxilla; mt, maxillary teeth; pa, palatine; pbs, parabasisphenoid; pt, pterygoid; qd, quadrate; fl, flange
